# Duration of untreated bipolar disorder: a multicenter study

**DOI:** 10.1038/srep44811

**Published:** 2017-03-22

**Authors:** Ling Zhang, Xin Yu, Yi-Ru Fang, Gabor S. Ungvari, Chee H. Ng, Helen F. K. Chiu, Hui-Chun Li, Hai-Chen Yang, Qing-Rong Tan, Xiu-Feng Xu, Gang Wang, Yu-Tao Xiang

**Affiliations:** 1 The National Clinical Research Center for Mental Disorders, China & Center of Depression, Beijing Institute for Brain Disorders & Mood Disorders Center, Beijing Anding Hospital, Capital Medical University, Beijing, China; 2 Unit of Psychiatry, Faculty of Health Sciences, University of Macau, Macao SAR, China; 3 Peking University Institute of Mental Health (the sixth Hospital) & National Clinical Research Center for Mental Disorders & the key Laboratory of Mental Health, Ministry of Health (Peking University), Beijing, China; 4 Division of Mood Disorders, Shanghai Mental Health Center, Shanghai Jiao Tong University School of Medicine, Shanghai, China; 5 The University of Notre Dame Australia/Marian Centre, Perth, Australia; 6 School of Psychiatry & Clinical Neurosciences, University of Western Australia, Perth, Australia; 7 Department of Psychiatry, University of Melbourne, Melbourne, Victoria, Australia; 8 Department of Psychiatry, Chinese University of Hong Kong, Hong Kong SAR, China; 9 The Second Affiliated Hospital, College of Medicine, Zhejiang University, Hangzhou, Zhejiang province, China; 10 Division of Mood Disorders, Shenzhen Mental Health Centre, Shenzhen, Guangdong province, China; 11 Department of Psychiatry, Xijing Hospital, Fourth Military Medical University, Xi’an, Shannxi province, China; 12 Department of Psychiatry, the First Affiliated Hospital of Kunming Medical University, Kunming, Yunnan province, China

## Abstract

Little is known about the demographic and clinical differences between short and long duration of untreated bipolar disorder (DUB) in Chinese patients. This study examined the demographic and clinical features of short (≤2 years) and long DUB (>2 years) in China. A consecutively recruited sample of 555 patients with bipolar disorder (BD) was examined in 7 psychiatric hospitals and general hospital psychiatric units across China. Patients’ demographic and clinical characteristics were collected using a standardized protocol and data collection procedure. The mean DUB was 3.2 ± 6.0 years; long DUB accounted for 31.0% of the sample. Multivariate analyses revealed that longer duration of illness, diagnosis of BD type II, and earlier misdiagnosis of BD for major depressive disorder or schizophrenia were independently associated with long DUB. The mean DUB in Chinese BD patients was shorter than the reported figures from Western countries. The long-term impact of DUB on the outcome of BD is warranted.

Bipolar disorder (BD) is a severe and chronic mental illness^1^ with the lifetime prevalence of BD type I (BD-I) and type II (BD-II) being approximately 1.1% and 1.6%, respectively^2^. BD is often unrecognized or misdiagnosed. As a consequence, BD has a long untreated period^3^,^4^,^5^,^6^, resulting in poor clinical outcomes including frequent recurrence and hospitalizations, suicidal behavior, high risk of rapid cycling and poor functional outcomes^4^,^6^,^7^.

Over the past decades several studies have been conducted in Western countries focusing on the duration of untreated illness (DUI) defined as the interval between the onset of psychiatric disorders and the first treatment received^8^. Commonly reported demographic and clinical correlates of DUI included BD-II, female gender, early onset, depressive onset, long duration of illness, suicide attempts, family history of BD and co-morbid anxiety disorders^3^,^4^,^7^,^9^. Recently the concept of duration of untreated bipolar disorder (DUB) has been recommended to replace DUI^4^,^7^ to ensure a more accurate definition.

The prevalence and clinical features of mood disorders are affected by a host of biological and socio-cultural factors, therefore findings obtained in Western settings are unlikely to be applicable to other parts of the world^10^. Research findings in the Western world do not cover the full range of mood symptoms experienced by Chinese patients^11^,^12^,^13^,^14^,^15^, which gave the impetus to conduct this study.

This study set out to examine the demographic and clinical features of Chinese patients with short (≤2 years) and long (>2 years) DUB.

## Methods

### Study participants and settings

This was a cross-sectional, multicenter study (Registration number: NCT01770704) initiated by the Chinese Society of Psychiatry conducted in seven major psychiatric hospitals/units located in the north, south, east, west and central parts of China representing a range of clinical settings. Both inpatients and outpatients were consecutively screened during the study period (between January, 2013 and January, 2014) with the following study entry criteria: age 18 years or older, diagnosis of BD-I or BD-II according to DSM-IV criteria^16^ established by two consultant psychiatrists with over 15 years clinical experience, at least one depressive or manic episode during the past year, understanding of the aims of the study and the contents of the clinical interview and willingness to provide informed consent. The study protocol was approved by the Clinical Research Ethics Committees of Beijing Anding Hospital, Peking University Institute of Mental Health, Shanghai Mental Health Center, the Second Affiliated Hospital of Zhejiang University, Shenzhen Mental Health Centre, Xijing Hospital and the First Affiliated Hospital of Kunming Medical University, China. The protocol including the methods was performed in accordance with the Declaration of Helsinki and the relevant ethical guidelines and regulations in China. Informed consents were obtained from all participants.

### Assessment procedure

All patients with a diagnosis of BD receiving treatment in the participating hospitals/units were invited to take part in the study and if agreed, they were referred by their treating psychiatrists to the investigators. Patients satisfying the above entry criteria were invited to participate in the study. Patients’ basic demographic and clinical data were collected with a form designed for the study in the course of a clinical interview supplemented by a review of the medical records. DUB was defined as the interval between the onset of the first mood episode and the first treatment with a mood stabilizer^4^,^5^. Mood stabilizers prescribed included lithium, valproate, carbamazepine and lamotrigine. There is no widely accepted gold standard threshold for defining short/long DUB. Following earlier studies^3^ and the recommendation of the Chinese Society of Psychiatry Expert Committee, in this study the 2-year cutoff point differentiated between short (≤2 years) and long DUB (>2 years).

### Statistical analysis

The data were analyzed using SPSS Version 21.0 for Windows. Comparison of the socio-demographic and clinical characteristics between the short and long DUB groups was made using independent sample t-test, Mann-Whitney U test, and chi-square test, as appropriate. Multiple logistic regression analysis with the “Enter” method was used to determine the demographic and clinical variables that were independently associated with long DUB. Long DUB was the dependent variable, while variables that significantly differed between the two groups in the above univariate analyses were entered as independent variables. The one-sample Kolmogorov-Smirnov test was used to check the normality of the distribution of the continuous variables. The level of significance set at 0.05 (two-tailed).

## Results

Altogether 555 patients were invited to participate in the study; 35 (6.3%) refused thus 520 (93.7%) fulfilled the study entry criteria and formed the study sample. The mean DUB was 3.2 ± 6.0 years. Long DUB accounted for 31.0% of the sample. Table 1 presents the basic demographic and clinical characteristics for the whole sample and separately for patients with short and long DUB. Patients in the long DUB group were older, had a longer duration of illness, more family history of mood disorders, more likely to have BD-II and an earlier misdiagnosis of BD for major depressive disorder or schizophrenia.

Multivariate analyses revealed that longer duration of illness (p < 0.001, Odds ratio = 1.1, 95% CI = 1.1–1.2), diagnosis of BD-II (p = 0.01, Odds ratio = 2.0, 95% CI = 1.2–3.6), earlier misdiagnosis of BD for major depressive disorder (p < 0.001, Odds ratio = 4.1, 95% CI = 2.4–7.3) or schizophrenia (p < 0.001, Odds ratio = 7.4, 95% CI = 3.8–14.4) were independently and positively associated with long DUB, which accounted for a maximum of 42.7% of the model (p < 0.001) (Table 2).

## Discussion

To the best of our knowledge, this was the first multicenter study in China that compared the demographic and clinical features between BD patients with short and long DUB. The main finding is that Chinese BD patients had a mean DUB of 3.2 years; 31.0% of the sample had long DUB. Long DUB was significantly associated with longer duration of illness, diagnosis of BD type II, and earlier misdiagnosis of BD for major depressive disorder or schizophrenia. The rate of participation in the study was high, reflecting a good relationship between patients and their treating psychiatrists. Respect for the medical profession is still rather high in China – although it is being eroded – and patients are unlikely to refuse a request from their doctors.

The mean DUB in this study was shorter than figures of 6.7–20 years reported from Western countries^3^,^4^,^5^,^7^,^17^, but similar to the finding of 3.9 years reported from China^18^. The different definitions of DUB may partly account for the inconsistency of the results. For example, studies used the interval between the first symptoms and diagnosis, but others referred to the time between the first episode and any treatment or prescription of mood stabilizers in defining DUB^4^. Furthermore, there were more patients with BD-I than with BD-II in this sample (76.7% vs 23.3%), which would shorten DUB as BP-I patients are a great deal easier to identify^3^,^19^. In addition, the discrepancy in DUB between studies may be due to the differences in sampling methods, the distribution of bipolar subtypes and the type diagnostic assessment. All participating psychiatric units or hospitals in this study are located in major cities thus patients could easily access mental health services. In addition, psychiatrists in major centres are usually better trained to ascertain BD resulting in relatively short DUB.

In multivariate analyses DUB was independently associated with several clinical variables. Patients with longer DUB had a longer duration of illness, which is consistent with earlier findings^3^. BD-II has been a major contributing factor to long DUB^4^,^19^,^20^, which is also confirmed in this study. Hypomania is often perceived as normal condition by patients and their families, therefore it is not spontaneously reported^21^,^22^. Furthermore, depressive episodes usually last longer than hypomania in BD-II, thus patients seek help for depression more often than for hypomania^23^. Clinicians sometimes overlook the history of hypomania if patients present with a depressive episode. All these factors contribute to the failure to diagnose BD-II leading to an extended DUB.

Multiple episodes of major depression frequently occur prior to the first episode of hypomania or mania^24^,^25^. In addition, depressive phases occur more frequently than hypomanic or manic phases^26^. Therefore BD is frequently misdiagnosed for major depressive disorder^21^,^25^,^27^, which, in turn, leads to longer DUB as found in this study. Similarly, BD could manifest initially with psychotic symptoms prior to the first signs of mania that would suggest the diagnosis of schizophrenia, which is another reason for longer DUB^28^. This is consistent with the finding of this study. Early onset of BD (≤21 years) was reported to be a risk factor for long DUB^4^,^29^,^30^,^31^,^32^, but this was not confirmed in this study.

The major strength of this study is its large, multi-center sample. However, the results should be interpreted with caution due to several methodological limitations. First, similar to most studies, some of the data were collected retrospectively, such as onset age and that of the first episode, thus potential recall bias could not be avoided. Due to logistical reasons, some important variables, such as genetic and neuroimaging data, could not be collected. Second, due to the cross-sectional study design, the causality between DUB and other variables could not be examined. Third, second-generation antipsychotics (SGAs) show certain mood-stabilizing properties. Considering that the purpose of the SGA prescription were difficult to identify in cross-sectional surveys, only lithium, sodium valproate, carbamazepine and lamotrigine were classified as mood stabilizers. Fourth, there is no widely accepted consensus regarding the definition of short/long DUB. Finally, following earlier studies^4^,^5^ prescription of mood stabilizers identified DUB although mood stabilizers do not necessarily indicate the diagnosis of BD.

In conclusion, the mean DUB in Chinese BD patients was shorter than the figures in the literature. The differences between long and short DUB with respect to Chinese BD patients’ demographic and clinical characteristics were consistent with those found in Western clinical settings. Prospective studies examining the long-term clinical outcome of DUB are warranted.

## Additional Information

**How to cite this article:** Zhang, L. *et al*. Duration of untreated bipolar disorder: a multicenter study. *Sci. Rep.* **7**, 44811; doi: 10.1038/srep44811 (2017).

**Publisher's note:** Springer Nature remains neutral with regard to jurisdictional claims in published maps and institutional affiliations.

## Figures and Tables

**Table 1:** Demographic and clinical characteristics of the sample.

Variables	The whole sample (n = 520)	DUB ≤ 2 years (n = 359)	DUB > 2 years (n = 161)	*P*-value
Male, n (%)	252 (48.5)	176 (49.0)	76 (47.2)	0.70
Employed, n (%)	290 (55.8)	202 (56.3)	88 (54.7)	0.73
Living with family, n (%)	478 (91.9)	331 (92.2)	147 (91.3)	0.73
Family history of mood disorders, n (%)	70 (13.5)	38 (10.6)	32 (19.9)	0.004
Age at onset, n (%)				0.24
Early onset (≤21 yrs)	181 (34.8)	119 (33.1)	62 (38.5)	
Intermediate onset (21–37 yrs)	232 (44.6)	169 (47.1)	63 (39.1)	
Late onset (>37 years)	107 (20.6)	71 (19.8)	36 (22.4)	
BD type, n (%)				0.005
Type I	399 (76.7)	288 (80.2)	111 (68.9)	
Type II	121 (23.3)	71 (19.8)	50 (31.1)	
Polarity of first mood episode, n (%)				0.12
Depressive	307 (59.0)	204 (56.8)	103 (64.0)	
Manic/hypomanic/mixed	213 (41.0)	155 (43.2)	58 (36.0)	
History of misdiagnosis, n (%)				
Major Depressive Disorder	270 (51.9)	166 (46.2)	104 (64.6)	<0.001
Schizophrenia	89 (17.1)	39 (10.9)	50 (31.1)	<0.001
Anxiety disorders	39 (7.5)	23 (6.4)	16 (9.9)	0.16
Lifetime suicide attempts, n (%)	54 (10.4)	35 (9.7)	19 (11.8)	0.48
Comorbid substance abuse, n (%)	36 (6.9)	28 (7.8)	8 (5.0)	0.24
	**Mean** ± **SD**	**Mean** ± **SD**	**Mean** ± **SD**	***P-*****value**
DUB (years)	3.2 ± 6.0	0.3 ± 0.7	9.6 ± 7.7	—
Age (years)	35.1 ± 13.2	32.8 ± 12.4	40.3 ± 13.6	<0.001*
Education (years)	13.1 ± 3.4	13.2 ± 3.3	12.8 ± 3.6	0.41
Duration of illness (years)	6.4 ± 8.3	3.8 ± 6.2	12.3 ± 9.4	<0.001*
Number of admissions after the diagnosis of BD	1.9 ± 1.5	2.0 ± 1.6	1.8 ± 1.3	0.13*

^*^Mann-Whitney U test. BD = bipolar disorder; DUB = duration of untreated bipolar disorder.

**Table 2:** Demographic and clinical characteristics independently associated with long DUB by multiple logistic regression analysis.

	*P* value	Odds ratio	95% C. I.
Age (years)	0.44	1.0	0.9–1.02
Duration of illness (years)	<0.001	1.1	1.1–1.2
BD-II	0.01	2.0	1.2–3.6
A history of misdiagnosis as major depressive disorder	<0.001	4.1	2.4–7.3
A history of misdiagnosis as schizophrenia	<0.001	7.4	3.8–14.4
Family history of mood disorders	0.35	1.3	0.7–2.5

Study sites were controlled for. Total model: P < 0.001, R^2^ = 0.427.
